# Wearable Multi-Sensor Positioning Prototype for Rowing Technique Evaluation

**DOI:** 10.3390/s24165280

**Published:** 2024-08-15

**Authors:** Luis Rodriguez Mendoza, Kyle O’Keefe

**Affiliations:** Geomatics Department, Schulich School of Engineering, University of Calgary, 2500 University Drive NW, Calgary, AB T2N 1N4, Canada; kpgokeef@ucalgary.ca

**Keywords:** rowing, ultra-wideband (UWB), GNSS, inertial sensors (IMU), inertial navigation system (INS), wearable technology

## Abstract

The goal of this study is to determine the feasibility of a wearable multi-sensor positioning prototype to be used as a training tool to evaluate rowing technique and to determine the positioning accuracy using multiple mathematical models and estimation methods. The wearable device consists of an inertial measurement unit (IMU), an ultra-wideband (UWB) transceiver, and a global navigation satellite system (GNSS) receiver. An experiment on a rowing shell was conducted to evaluate the performance of the system on a rower’s wrist, against a centimeter-level GNSS reference trajectory. This experiment analyzed the rowing motion in multiple navigation frames and with various positioning methods. The results show that the wearable device prototype is a viable option for rowing technique analysis; the system was able to provide the position, velocity, and attitude of a rower’s wrist, with a positioning accuracy ranging between ±0.185 m and ±1.656 m depending on the estimation method.

## 1. Introduction

The objective of most competitive rowing is to complete a 2000 m course in the fastest time. Each stroke must be efficient, and each phase must be executed to perfection. The stroke is a movement that goes through four main phases, the catch, the drive, the finish, and the recovery. Rowing technique has been widely researched and evaluated based on the rowing stroke [1,2,3,4,5]. Baudouin and Hawkins concluded that the propulsive force, that directly affects boat velocity, occurs at the oar blade, which is affected by the force and movement of the rower at the handle [5]. Therefore, looking at the oar handle neglects the athlete’s anthropometrics and analyzes only the quality of the stroke. Past research has demonstrated that oar handle kinematics correlate with the rower’s technique and skill level [1,4]. Handle kinematics have been measured in terms of stroke length, stroke rate, handle velocity, and handle acceleration [4,5,6,7,8,9,10,11]. However, handle positioning is an area that has not been widely explored for estimating these metrics. Additionally, in crew boats, handle position is of great relevance because it can be used to determine the synchrony of the athletes [6,12,13,14].

Inertial sensors are the most common type of devices used to measure the kinematics of handles in rowing [2,7]. A triad of inertial sensors coupled with an estimator is called an inertial navigation system (INS) [15]. In the context of sport, an INS is often used to estimate kinematic parameters that represent the orientation of a body segment or sport equipment in an inertial frame [16]. Therefore, using an INS to track the motion of the oar handle during the rowing motion is an appropriate approach. However, an INS solution degrades with time due to sensor biases and process errors because it integrates accelerations and angular rates to determine velocity, position, and attitude [15]. Specifically for rowing, one study used inertial and surface electro-myography sensor networks placed on the body of an athlete to identify the muscle activity and acceleration at each stroke phase [6]. This method yielded an understanding of muscle recruitment sequencing and their correlation with the stroke cycle [6]. Moreover, it allowed for athlete synchronization analysis in crew boats [6]. However, this depends on sensor placement and rower’s anthropometrics and does not evaluate the motion of the handle. Oar kinematics provide important information about rowers’ technique. Reference [4] developed a sensor network consisting of three IMUs; two of these sensors were placed on the oars near to the oarlock, and the last one was placed at the middle of the boat in front of the rower. Reference [4] assessed the technique of 18 rowers based on stroke rate, stroke length, recovery/drive ratio, and feathered/squared blade ratio. Most of the metrics for the results are based on the oar angles measured by the inertial sensors. The results demonstrated that IMUs can be used to obtain important information about oar kinematics. The same research group published two other studies relevant to rowing technique analysis based on inertial sensors [10,11]. In [10], a mobile phone was strapped onto an oar to evaluate stroke length based on the oar angle measured on the phone’s IMU and comparing the oar angle to a reference trajectory obtained from a potentiometer [10]. Furthermore, in [11], an IMU was placed inside an oar (instead of a mobile phone) and measured the oar orientation throughout a stroke using an integration algorithm; the model was validated with a reference trajectory obtained from a potentiometer located on the oarlock. This series of studies revealed that inertial sensors can be used in many ways to evaluate rowing technique, especially when placed on the oar. However, none of these papers evaluated the position of the handle, showing a gap in the literature that this article will address. For applications in pedestrian navigation (i.e., running and jogging), pedestrian dead reckoning (PDR) is a method that has been used to improve the navigation performance of low-cost INS based on the periodic motion of the human gait [15]. In addition to PDR, INS errors are typically controlled with external aiding sources of position and velocity, such as GNSS and UWB [17]. Moreover, PDR has been adapted to sports with periodic motions such as cycling [18]; thus, a similar approach is explored for rowing in this study called rowing dead reckoning (RDR).

Moreover, in rowing, radio-frequency-based sensors, such as GNSS and UWB, have not been implemented in technique analysis nor used to control INS errors [2]. GNSS has been used only to track the position and velocity of boats during competitions and training [19,20,21]. Differential GNSS (DGNSS) has been used as a reference trajectory to validate positioning systems and models [22,23]. Carrier-phase DGNSS can achieve centimeter-level accuracy in open-sky conditions [15], thus making it an appropriate method for validating positioning results. Ultra-wideband (UWB) ranging is a method used for indoor and outdoor localization. Double sided two-way ranging (DS-TWR) is a process that requires the exchange of four messages between a tag and an anchor to estimate the time of arrival (TOA) between messages. Using this process avoids the need for clock and frequency synchronization between tag and anchor [24]. In this paper, a DS-TWR-TOA method is used to obtain distance measurements between two anchors and a single tag. Our previous work studied the validity of UWB to track handle motion indoors on a rowing machine [24]. The system achieved an accuracy of ±0.21m using a periodic extended Kalman filter (PEKF) in a two-dimensional frame [24]. This paper further explores the system and the model in an outdoor setting and expands the solution to a three-dimensional frame. This study determines whether a wearable positioning system prototype (WPP) that uses UWB ranging, inertial sensors, and a GNSS receiver is a viable tool for rowing technique analysis based on the positioning of the oar handle during the rowing motion. This paper proposes and compares five solutions: UWB, standalone INS, RDR INS, GNSS-aided INS, and UWB-aided INS using a carrier-phase DGNSS as a reference.

The goal of this study is to show that the WPP can fill the gap in the literature by providing information of the position of the oar handle, while demonstrating that radio-frequency technologies such as UWB and GNSS can be used as an accurate source of technique evaluation in rowing. The remainder of this paper is organized as follows: Section 2 describes the WPP and its specifications and covers the mathematical models and algorithms implemented to obtain the handle position in three dimensions. Section 3 shows the evaluation and results using each of the methods, and Section 4 provides conclusions and recommendations.

## 2. Materials and Methods

### 2.1. System Description

**Figure 1:**
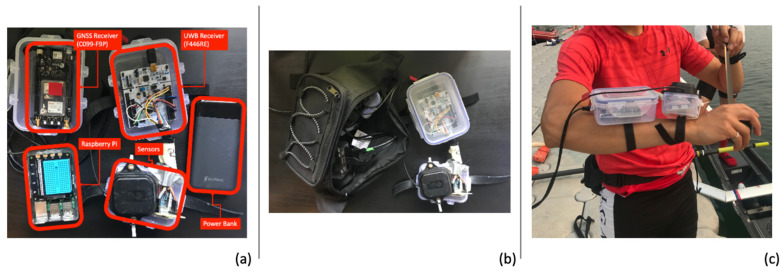
Wearable positioning prototype components.

The WPP consists of two sensor modules, one to access the output of the GNSS data and the other for the UWB and inertial measurement unit (IMU) measurements. The data are transferred in parallel to a logging personal computer (PC). The entire system is powered by a 5V USB power bank. Figure 1 shows the WPP’s components ((a) all components, (b) components ready to wear, (c) components on rower) and Table 1 shows the sensor specifications.

The microcontrollers used for the sensor modules are a C099-F9P and a NUCLEO-F446RE [25,26]. They were selected because the C099-F9P was specifically designed by *U-Blox* to easily access GNSS data from the ZED-F9P chip [27] while the NUCLEO-F446RE has available connections which allow for high data rate transmission between UWB radios and reception of IMU measurements simultaneously.

A *Raspberry Pi* (RPi) with a Pimoroni Explorer Hat (PEH) was selected as the logging computer due to its compactness and low power requirements. The RPi is controlled using *VNC Viewer*, which is a remote desktop application. The purpose of using a remote desktop is to eliminate additional hardware (i.e., a screen, mouse, and keyboard) and easy monitoring of the logging process while the user is wearing the WPP. The addition of the PEH is to allow the user to start and stop the logging functions with touch buttons [28].

The output is two log files, one for each module. The UWB/IMU file is a text file with the information of the source (i.e., transmitter ID), the distance observations from the UWB ranging, and the IMU measurements. The GNSS module outputs a customized binary format that can be transformed into a receiver independent exchange (RINEX) format and then processed using the open-source software RTKLIB version 2.4.2 [29].

### 2.2. Methods

**Figure 2:**
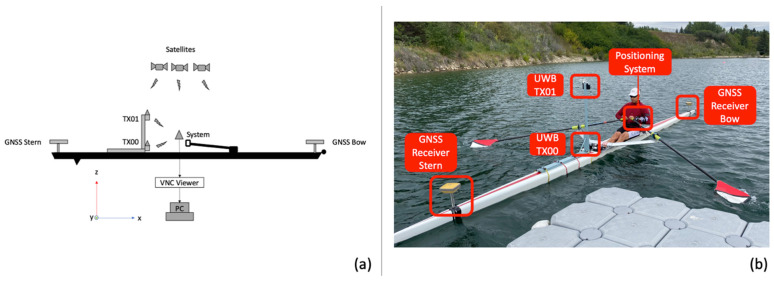
(**a**) Boat setup schematic. (**b**) Boat setup on water.

Oar handle kinematics can be described in terms of position, velocity, and attitude angles, expressed in a three-dimensional space. The combination of these terms is often referred to as a navigation solution [30]; it is possible to represent this solution in different coordinate frames. This section introduces the navigation frames used in this study, an overview of attitude angles and coordinate transformations, the mathematical models for processing ultra-wideband ranging measurements, three algorithms used for inertial navigation, and the experimental setup. Figure 2a,b show the boat setup schematic showing the placement of the components with respect to the boat coordinate system.

#### 2.2.1. Body Frame

The sensor or body frame aligns with the axes of the moving object [31]. This study assumes that the origin of the sensor frame (GNSS antenna, UWB antenna, and IMU origin) coincides with the center of gravity of the sensor device which in this case is mounted to the wrist of the rower. This is represented with the superscript and the subscript denoted as ${\left[■\right]}_{b}^{b}$.

##### East-North-Up (ENU)

The ENU frame is a local-level or navigation frame [31]. The general representation of a navigation frame is described with the superscript and the subscript denoted as ${\left[■\right]}_{n}^{n}$. In this paper, ENU uses the World Geodetic System (WGS84) as the reference model for the Earth and has its origin determined by the location of a GNSS base station a few hundred meters away. The coordinate system has the *x*-axis pointing in the direction of the east, the *y*-axis towards the true north, and the *z*-axis in the up direction.

##### Boat Frame (XYZ)

The boat frame has an origin determined in front of the rower on the rowing shell. The boat frame has the *x*-axis pointing in the direction along the boat towards the bow (back of the rower), the *y*-axis towards the starboard (left of the rower), and the *z*-axis in the vertical direction.

For inertial navigation, it is important to consider the properties of the coordinate systems. The ENU frame is a quasi-inertial frame that allows for inertial navigation. However, the XYZ frame is not inertial; this is because the boat is neither stationary nor moving at a constant speed and instead accelerates and decelerates during different phases of each stroke. Therefore, in order to obtain an inertial navigation solution, this paper determined a boat frame that is instantaneously coincidental with the ENU frame at a moment where it is assumed the rower and oar are momentarily not moving (i.e., translating or rotating), thus creating a static frame for inertial navigation per stroke.

##### Attitude Angles and Coordinate Transformations

It is possible to transform one system to another one by carrying out a rotation about each of the three rotational axes [31].

Roll (ϕ) is the angle between the body’s *y*-axis ($y^{b}$) and the horizontal plane.

Pitch (θ) is the angle between the body’s *x*-axis ($x^{b}$) with the horizontal plane.

Azimuth, yaw, or heading (ψ) is the difference between the forward axis with respect to the north in ENU or the along track for the boat frame. Azimuth also represents the rotation angle about the body’s *z*-axis ($z^{b}$). The three terms are used interchangeably in this paper.

The relationship between the navigation frame and the body frame can be described with a transformation matrix $R_{n}^{b}$. The rotation sequence in this paper is pitch-roll-azimuth [32]. To do the opposite transformation, the transpose of the rotation matrix is used [30,31].

(1) $$R_{n}^{b}=\left[\begin{array}{c} \cos\psi \cos\theta -\sin\psi \sin\theta \sin\phi \\ -\sin\psi \cos\phi \\ \cos\psi \sin\theta +\sin\psi \sin\phi \cos\theta \end{array}\;\begin{array}{c} \sin\psi \cos\theta +\cos\psi \sin\theta \sin\phi \;\\ \cos\psi \cos\phi \\ \sin\psi \sin\theta -\cos\psi \sin\phi \cos\theta \end{array}\;{\begin{array}{c} -\sin\theta \cos\phi \;\\ \sin\phi \\ \cos\theta \cos\phi \end{array}}^{\;}\right]$$

#### 2.2.2. UWB Positioning Models

The models presented in this study are based on parametric least squares (LS) and the extended Kalman filter (EKF). These models include trilateration, periodic least squares (PLS), constant velocity EKF, and periodic EKF (PEKF). These models were validated for indoor rowing handle tracking in our previous work [24].

Importantly, trilateration gives a single position estimate per epoch that is used as the input of PLS, constant velocity EKF, and PEKF. Moreover, both the constant velocity EKF and PEKF are filtered versions of the trilateration model; however, PEKF uses the solution from PLS as an initialization method.

##### Trilateration

The WPP receives two UWB ranging measurements at every epoch, one from each transmitter placed in front of the rower. From the boat setup and geometry shown in Figure 2a, having two observations and two unknowns gives a unique two-dimensional solution (*x*- and *z*-axis). However, the motion is a three-dimensional movement. Therefore, a range constraint (constant distance measurement) from the oarlock to the WPP is added to provide a third observation and obtain a three-dimensional solution.

Each range measurement can be expressed as a function of the known and unknown positions:

(2) $$f\left(x\right)=\sqrt{{\left(x_{i}^{tx}-x^{rx}\right)}^{2}+{\left(y_{i}^{tx}-y^{rx}\right)}^{2}+{\left(z_{i}^{tx}-z^{rx}\right)}^{2}}$$

where $x_{i}^{tx}$, $y_{i}^{tx}$, and $z_{i}^{tx}$ are the X-, Y-, and Z-coordinates of the transmitter (or oarlock) *i*, respectively, and $x_{i}^{rx}$, $y_{i}^{rx}$, and $z_{i}^{rx}$ are the unknown coordinates of the WPP.

The geometry of the WPP and the transmitters contributes to the accuracy and precision of the trilateration solution, and each set of estimated coordinates is unique. Our previous work showed that the system achieved an accuracy of ±0.21 m in a two-dimensional frame [24].

##### Periodic Least Squares (PLS)

PLS is a nonlinear model with a function f(x,t) that describes a periodic wave that models the handle motion during the rowing stroke. This model is based on the work of [33,34,35] and receives the position estimates of trilateration as input. The number of measurements that represent a stroke cycle varies depending on the stroke rate. A fixed number is manually selected for the first stroke cycle of each test.

The function returns a specific waveform for each axis, and it can be represented in polar coordinates as

(3) $$f\left(x,t\right)=A_{0}+A_{1}\cos\left(\omega t+\phi_{1}\right)+A_{2}\cos\left(2\omega t+\phi_{2}\right)$$

where $A_{0}$ is the direct current (DC) offset, $A_{1}$ is the first harmonic amplitude, ω is the angular frequency, t is the time, $\phi_{1}$ is the first harmonic phase angle, $A_{2}$ is the second harmonic amplitude, and $\phi_{2}$ is the second harmonic phase angle. Thus, the parameter vector ($\hat{x}$) becomes

(4) $$\hat{x}={\left[A_{0}\;A_{1}A_{2}\phi_{1}\;\phi_{2}\;\omega \right]}^{T}$$

The Jacobian matrix of (5) forms the design matrix $H_{k+1}$ and can be written at time *k*+1 as

(5) $$H_{k+1,1}=1\;H_{k+1,2}=\cos\left(\omega t_{k}+\phi_{1}\right)\;H_{k+1,3}=\cos\left(2\omega t_{k}+\phi_{2}\right)\;H_{k+1,4}=-A_{1}\sin\left(\omega t_{k}+\phi_{2}\right)\;H_{k+1,5}=A_{2}\sin\left(2\omega t_{k}+\phi_{2}\right)\;H_{k+1,6}=-A_{1}t_{k}\sin\left(\omega t_{k}+\phi_{1}\right)-2A_{2}t_{k}\sin\left(2\omega t_{k}+\phi_{2}\right)$$

This method estimates the position of the handle throughout a full cycle (stroke) independently for each axis (i.e., each axis has independent parameter vectors including frequency).

##### Extended Kalman Filter (EKF) Overview

**Figure 3:**
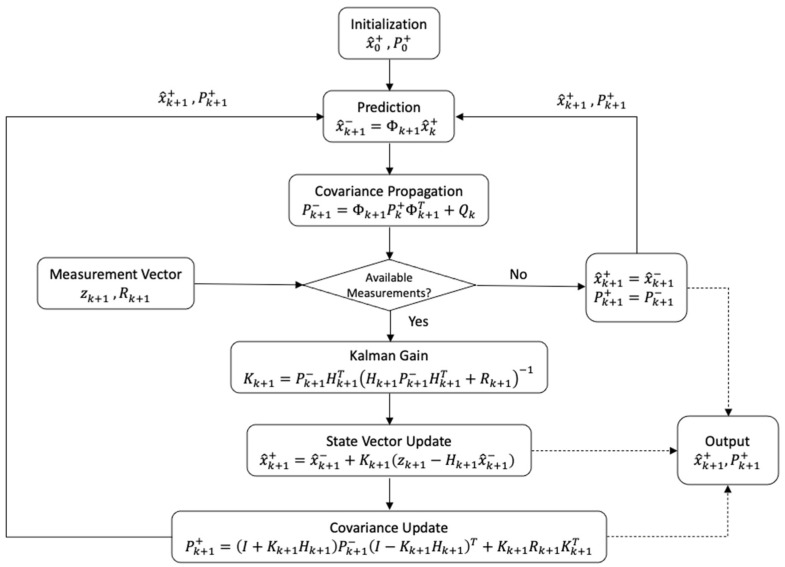
Discrete Kalman filter flowchart.

EKF is an iterative estimation method for nonlinear functions that has been widely used in navigation. EKF extends the LS estimation with the prediction of the state vector, commonly known as the dynamic model [34,35,36,37]. The Kalman filter loop is shown in Figure 3.

##### Constant Velocity EKF

The EKF with constant velocity assumes that the object being tracked is moving at a constant speed. The state vector is formed by two parameters, position (*x*, *y*, *z*) and velocity (*v_x_*, *v_y_*, and *v_z_*):

(6) $${\hat{x}}_{k}={\left[x\;y\;z\;v_{x}\;v_{y}\;v_{z}\right]}^{T}$$

In the dynamic model, the state transition matrix $\Phi_{k+1}$ shows the relationship between time and distance to predict the velocity and position of the handle at time *k*:

(7) $$\Phi_{k+1}=\left[\begin{array}{cccccc} 1 & 0 & 0 & \Delta t & 0 & 0\\ 0 & 1 & 0 & 0 & \Delta t & 0\\ 0 & 0 & 1 & 0 & 0 & \Delta t\\ 0 & 0 & 0 & 1 & 0 & 0\\ 0 & 0 & 0 & 0 & 1 & 0\\ 0 & 0 & 0 & 0 & 0 & 1 \end{array}\right]$$

The time between epochs is determined as $\Delta t=t_{k}-t_{k+1}$. When the measurement vector $z_{k+1}$ is also formed by position and velocity, the design matrix $H_{k+1}$ is a *6 × 6* identity matrix.

##### Periodic EKF (PEKF)

PEKF is an extended version of PLS that includes a prediction in the algorithm. The filter is initialized with PLS for fast convergence. After the filter is initialized, the measurements from trilateration are the input.

The state vector ${\hat{x}}_{\;}$ and the design matrix $H_{k+1}$ in PEKF are the same as in Equations (4) and (6), and the transition matrix $\Phi_{k+1}$ is a 6 × 6 identity matrix. This dynamic model is selected because of the assumption that the best prediction is that the next stroke is similar to the previous stroke. This assumption is vulnerable to changes in frequency, but the addition of process noise allows the filter to place more weight on the new set of observations than on the prediction from the previous stroke.

#### 2.2.3. Strapdown Inertial Navigation System (INS)

An INS has three main components: an IMU, a pre-processing unit, and a mechanization module [31]. In strapdown systems, the sensors are rigidly mounted onto the body of the moving object. This study uses a low-cost micro-electromechanical system (MEMS) IMU formed by two tri-axial sensors: an accelerometer and a gyroscope. The mechanization module consists of a series of differential navigation equations that can be written in the local-level frame as follows [30,31]:

(8) $$\left({\begin{array}{c} {\dot{r}}^{l}\\ {\dot{V}}^{l}\\ \dot{R_{b}^{l}} \end{array}}^{\;}\right)=\left({\begin{array}{c} V^{l}\\ R_{b}^{l}f^{b}+g^{l}\\ R_{b}^{l}\Omega_{ib}^{b} \end{array}}^{\;}\right)$$

where ${\dot{[r}}^{l},\;{\dot{V}}^{l},{\dot{R}}_{b}^{l}{]}^{T}$ are the time derivatives of the navigation states: position, velocity, and attitude [31]. $f^{b}$ and $\Omega_{ib}^{b}$ are the specific force and skew-symmetric matrix of the angular rate measurements ($\omega^{b}$), respectively. $V^{l}$ is the velocity vector and $g^{l}$ is the normal gravity vector.

The mechanization module is initialized with a set of measurements ($f^{b}$ and $\omega^{b}$) and an initial alignment using attitude angles. The initial roll and pitch can be calculated using the accelerometer measurements when the device is at rest, with the following equations:

(9) $$\phi_{\;}=\tan^{-1}\left(\frac{f_{y}^{b}}{\sqrt{f_{x}^{b^{2}}+f_{z}^{b^{2}}}}\right)$$

(10) $$\theta_{\;}=\tan^{-1}\left(\frac{-f_{x}^{b}}{f_{z}^{b}}\right)$$

In this study, the azimuth is defined by the user because the MEMS-grade IMU does not have the ability to provide an orientation. A step-by-step description of the mechanization process can be found in [31].

#### 2.2.4. INS Loosely Coupled Integration

Loosely coupled integration is a cascade architecture where an aiding source is used to estimate sensor biases and errors from the mechanization process by introducing a Kalman filter [15,17,37,38].

**Figure 4:**
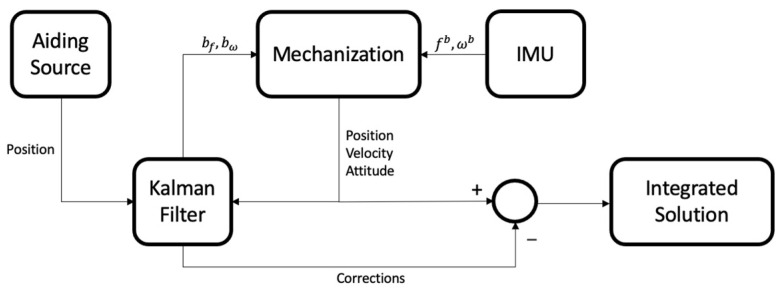
Loosely coupled integration flowchart.

The output of the loosely coupled integration is the corrected inertial navigation solution (i.e., position, velocity, and attitude) and the estimates of the sensor biases. This paper uses a closed-loop error-state Kalman filter algorithm. Figure 4 illustrates the loosely coupled integration process. The Kalman filter state vector can be described as

(11) $$\delta x=\left[{\begin{array}{c} \delta r_{eb}^{n}\\ \delta V_{eb}^{n}\\ \delta \Psi_{nb}^{n}\\ b_{f}\\ b_{\omega } \end{array}}^{\;}\right]$$

where $b_{f}$ and $b_{\omega }$ are the accelerometer and gyroscope biases, respectively. It is assumed that the bias states are modeled as first-order Gauss-Markov processes [31].

The state transition matrix can be written as

(12) $$\Phi_{k+1}=\left(\begin{array}{ccccc} I_{3\times 3} & I_{3\times 3}\Delta t & 0_{3\times 3} & 0_{3\times 3} & 0_{3\times 3}\\ 0_{3\times 3} & I_{3\times 3} & -\Omega \left({\hat{f}}_{ib}^{n}\right)\Delta t & {\hat{R}}_{b}^{n}\Delta t & 0_{3\times 3}\\ 0_{3\times 3} & 0_{3\times 3} & I_{3\times 3} & 0_{3\times 3} & {\hat{R}}_{b}^{n}\Delta t\\ 0_{3\times 3} & 0_{3\times 3} & 0_{3\times 3} & I_{3\times 3}-\beta_{b_{f}}\Delta t & 0_{3\times 3}\\ 0_{3\times 3} & 0_{3\times 3} & 0_{3\times 3} & 0_{3\times 3} & I_{3\times 3}-\beta_{b_{\omega }}\Delta t \end{array}\right)$$

where $\beta_{b_{f}}$ and $\beta_{b_{\omega }}$ are the diagonal matrices of the inverse of the correlation time (τ) for the sensor biases.

(13) $$\beta_{b_{f}}=\left(\begin{array}{c} \frac{1}{\tau_{b_{f_{x}}}}\\ 0\\ 0 \end{array}\;\begin{array}{c} 0\\ \frac{1}{\tau_{b_{f_{y}}}}\\ 0 \end{array}\;{\begin{array}{c} 0\\ 0\\ \frac{1}{\tau_{b_{f_{z}}}} \end{array}}^{\;}\right)\;\mathrm{and}\;\beta_{b_{\omega }}=\left(\begin{array}{c} \frac{1}{\tau_{b_{\omega_{x}}}}\\ 0\\ 0 \end{array}\;\begin{array}{c} 0\\ \frac{1}{\tau_{b_{\omega_{y}}}}\\ 0 \end{array}\;{\begin{array}{c} 0\\ 0\\ \frac{1}{\tau_{b_{\omega_{z}}}} \end{array}}^{\;}\right)\;$$

The measurement vector is the difference between the INS and the aiding source positions:

(14) $$z_{k+1}=r_{INS}-r_{Aiding}=\delta r$$

And the design matrix $H_{k+1}$ can be defined as follows:

(15) $$H_{k+1}=\left[I_{3\times 3}\;0_{3\times 3}\;0_{3\times 3}\;0_{3\times 3}\;0_{3\times 3}\right]$$

#### 2.2.5. Rowing Dead Reckoning (RDR)

We propose an analogous model to PDR to estimate the kinematics of oar handles that we call rowing dead reckoning (RDR). The first step of RDR is to identify a finish position (new stroke).

**Figure 5:**
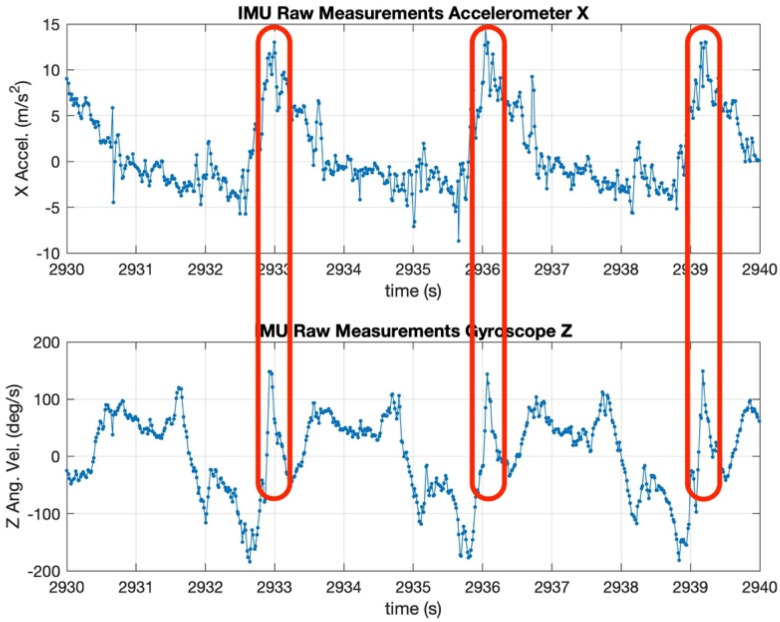
X accelerometer and Z gyroscope raw data.

At the finish position, the magnitude of the acceleration in the accelerometer’s *x*-axis is the largest (because the propulsion has ended and the boat is not decelerating). Similarly, the angular velocity on the gyroscope’s *z*-axis shows a well-defined peak (since the finish corresponds to a change in direction of the oar). In Figure 5, the two peaks are highlighted to show their correlation. This information is used to determine a new stroke and reset the initial conditions of the mechanization equations to minimize the accumulation of errors.

At the finish, it is assumed that the rower and oar are momentarily not moving (i.e., translating or rotating) in an instantaneous XYZ frame defined for the next stroke that coincides and aligns with the ENU frame at that instant.

RDR Stroke Detection Algorithm:

1.  **procedure** StrokeDetection (time (*t*), accelerometer ($f_{ib}^{b}$), gyroscope ($\omega_{ib}^{b}$))
2.  Define threshold for acceleration magnitude, *T_f_*
3.  Define threshold for angular velocity, *T_w_*
4.  Choose sliding window size, *N*
5.  Calculate mean values at time (*t*), $\bar{f_{ib}^{b}}\left(t\right)\;,\;\bar{\omega_{ib}^{b}}\left(t\right)$
6.  **If** { $\bar{f_{ib}^{b}}\left(t\right)>T_{f}\;\mathrm{and}\;\bar{\omega_{ib}^{b}}\left(t\right)>T_{\omega }\;\mathrm{and}\;\bar{f_{ib}^{b}}\left(t\right)>\bar{f_{ib}^{b}}\left(t-1\right)$} **then**
7.  **If** { t−(t−1)<1s} **then**
8.  **continue**
9.  End **if**
10. Declare new stroke at time (*t*)
11. End **if**
12. End **procedure**

The RDR stroke detection algorithm requires one to compute the mean values of sensor readings to reduce noise and prevent false stroke detections. Additionally, a time constraint of one-second separations between strokes is applied to prevent false detections. Then, mechanization equations from strapdown INS are applied. The orientation of the IMU at the instant that the recovery is detected, set to assumed values.

The next section describes the experimental setup for testing the WPP.

#### 2.2.6. Experiment

A test of the WPP was conducted at the Victoria City Rowing Club (VCRC) using a *Hudson* single scull, three *Topcon HiPer SR* multi-band GNSS receivers, and the proposed WPP. In Figure 2a,b, the U-blox GNSS receiver in the WPP serves both as an example of a low-cost wearable GNSS device (using single-frequency pseudo-ranges only) and a source for the reference trajectory (using dual-frequency carrier-phase observations).

Two of the *Topcon* receivers are located on the stern and on the bow, and the third is used as a base station for post-processing with a sampling rate of 10 Hz. The stroke rate maintained for this experiment was between 18 and 36 strokes per minute, meaning a maximum frequency of 0.6 Hz, well below the frequency of the sampling frequency of the reference trajectory. In terms of the UWB radios and the U-blox receiver, the sampling frequency was 50 Hz and 25 Hz, respectively.

The output of these receivers established the absolute position and orientation of the rowing shell and the transformation between the navigation and body frame of the shell. *RTKLIB* was used to post-process the data in carrier-phase DGNSS to obtain centimeter-level accuracy.

The carrier-phase DGNSS solution obtained in post-processing was used as the reference trajectory to evaluate the results obtained from the models presented in this paper. The expected accuracy from the *Topcon* receivers for DGNSS was 10 mm +0.8 ppm and 15 mm + 1.0 ppm for the horizontal and vertical axes (1σ), respectively [39]. For the WPP, the *U-blox* receiver specified an accuracy of 12 mm +1.2 ppm (1σ) for both the horizontal and vertical axes, also in carrier-phase DGNSS mode [40].

The UWB transmitters were placed on a customized stand facing the user, and the WPP was attached to the user’s left wrist and waist.

**Figure 6:**
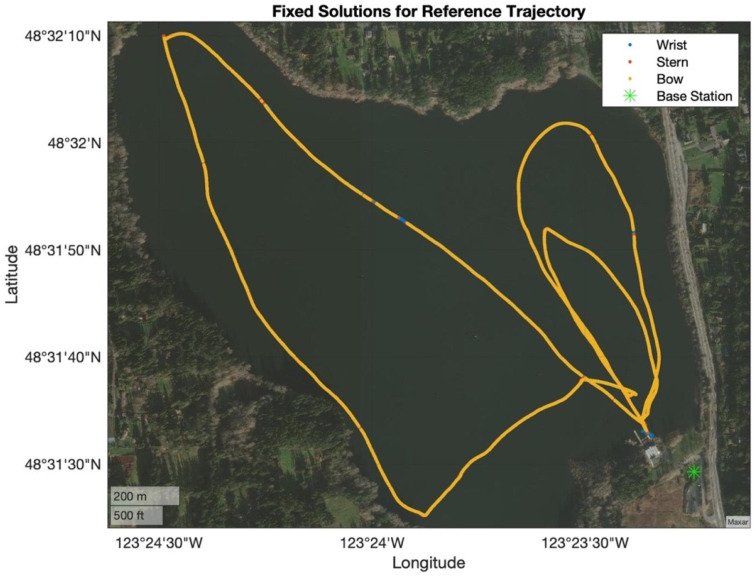
Experiment trajectory.

**Figure 7:**
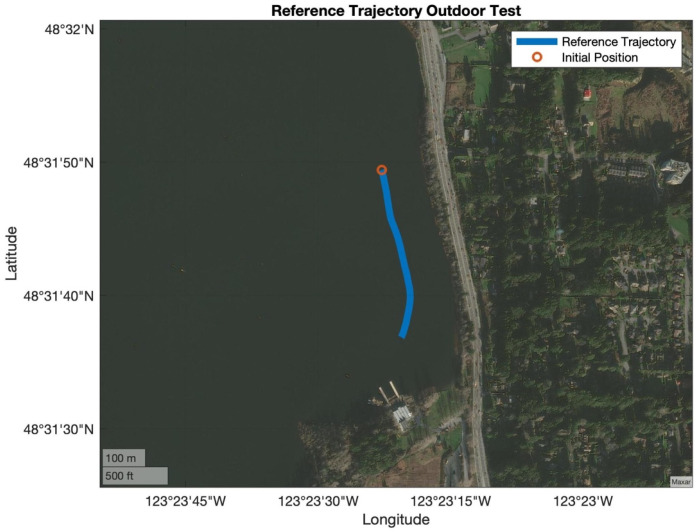
Detailed evaluation window.

Testing included rowing in various directions, speeds, stroke rates, and stroke lengths for approximately 90 min. Figure 6 shows the trajectory of the boat for the 90 min; from this figure, it is possible to observe that the trajectory was not in straight lines nor in the same location (i.e., back and forth). The two “small” loops on the eastern side of the lake were rowed in a clockwise direction, while the “large” loop on the western side of the lake was rowed in a counterclockwise direction. The speed of the boat and stroke length varied based on the side of the lake (i.e., water conditions), wind speed, and stroke rate. Figure 7 shows a subset of the test that was used to validate the models presented in this paper. This window was selected because the trajectory includes a section of rowing at a constant stroke rate, one section with an increase in rate (higher frequency), and one section with a decrease in rate (lower frequency). Additionally, the trajectory included a slight turn to the southwest. Recall that the purpose of this paper is to determine the feasibility of the WPP to be used as a technique analysis tool by providing information on the position of the handle based on UWB, GNSS, and INS with various mathematical models. The intention of this test was to evaluate common rowing patterns in a regular training session.

The next section presents the results obtained from the experimentation using the models shown in this section.

## 3. Results and Discussion

This section presents the results with each positioning model using a 120 s segment of the data that includes changes in direction, speed, stroke rate, and stroke length.

### 3.1. UWB Results

**Figure 8:**
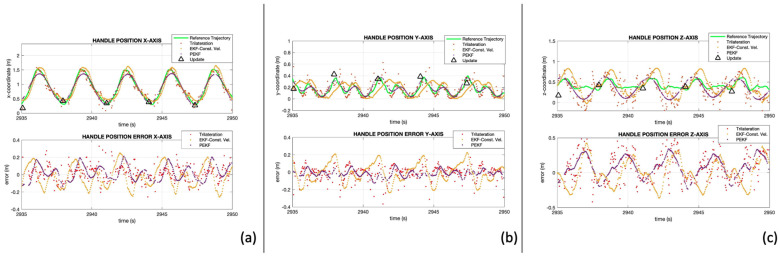
UWB positioning results, (**a**) X-coordinate, (**b**) Y-coordinate, (**c**) Z-coordinate in boat frame.

The accuracy of the models, ranges (TX00 ± 0.121 m, TX01 ± 0.106 m), and range constraint (±0.018 m) is evaluated using the GNSS reference trajectory. Figure 8 shows the position estimates for the X-, Y-, and Z-coordinates, respectively. Figure 8a–c contains the coordinate at the top and the error with respect to the reference at the bottom. The reference trajectory is represented with a green line, the trilateration is represented with red dots, the EKF with constant velocity is represented with yellow dots, the PEKF is represented with purple dots, and the time of update is represented with black triangles.

On the X-coordinate, PEKF reduced the variability of the trilateration solution and represented the handle position very accurately. The error is observed to be below ±0.20 m. The results from trilateration also represented the X-axis accurately but with some variance between estimates. Lastly, EKF with constant velocity overestimated the catch and the finish and returned the solution with the largest errors.

On the Y-axis, the handle motion shows two waves with different amplitudes at each stroke. The larger wave corresponds to the catch and the smaller one corresponds to the finish. PEKF represents both curves accurately. The results from trilateration follow the handle motion with a larger visible variance. EKF with constant velocity is not able to fully represent the two curves in the stroke. This is due to the rapid changes in velocities at the peaks and valleys from each curve. The largest observed errors for this model are within ±0.20 m, which is an acceptable accuracy in terms of positioning but not enough to represent the motion of handles for technique analysis.

On the Z-coordinate, the models can estimate a periodic curve; however, the amplitude is overestimated. This was expected as the accuracy of the ranges is approximately ±0.12 m and the amplitude of the motion is approximately 0.2 m. However, PEKF can be used to estimate the time of the catch, which is valuable for crew coordination and technique analysis.

PEKF outperformed trilateration and EKF with constant velocity. The output from this model clearly shows the motion of the handle and estimates the curves from each stroke on every axis.

### 3.2. INS Results

The roll and pitch angles used at the finish position are −38.5 deg and −24.9 deg, respectively. These angles were obtained through testing and manual adjustments.

The azimuth is assumed to be −45 deg; thus, computed angles about the Z-axis are with respect to an arbitrary start position rather than with respect to the forward axis of the boat.

The epoch-by-epoch DGNSS reference trajectory was numerically differentiated to obtain velocity and acceleration. The accuracy of the reference, from the carrier-phase DGNSS specifications, is an order or magnitude better the inertial solution and not affected by bias or drift.

**Figure 9:**
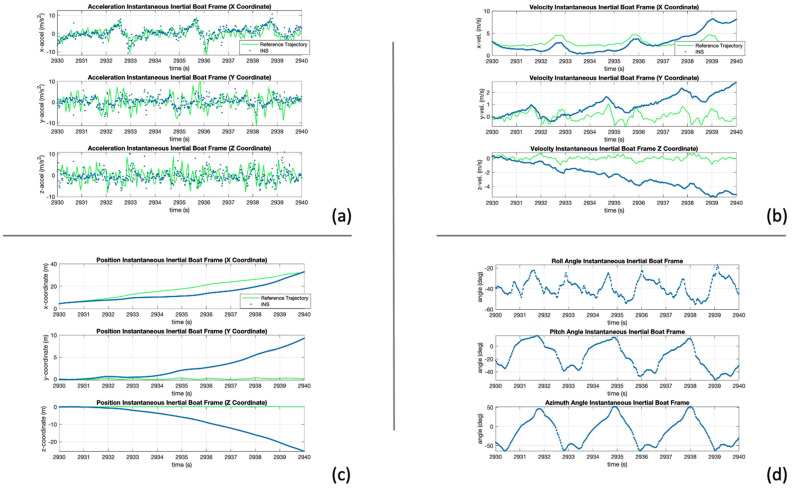
INS strapdown results, (**a**) acceleration, (**b**) velocity, (**c**) position, (**d**) attitude in instantaneous inertial boat frame.

Figure 9a shows the resulting acceleration of the oar handle in the instantaneous inertial XYZ frame. The acceleration is well aligned at the beginning of the test. However, it begins to drift over time. Some drift was expected due to the sensor biases; however, the bias effect on the attitude (Figure 9d) also influences the acceleration, thus affecting the velocity and position estimates.

Figure 9b shows the estimated velocity. All three axes are affected by the biases, especially on the Z-coordinate because some of the acceleration on the horizontal plane is transformed into this axis.

Lastly, Figure 9c shows the estimated position of the handle. The estimates on the horizontal plane show similar characteristics as the reference trajectory and begin to drift after some seconds. On the other hand, the effect of the biases and errors greatly influence the Z-coordinate and none of the expected characteristics are observable.

The results shown above demonstrated that standalone INS is not sufficient to estimate the position of the handle and requires an aiding method to reduce the effects of the sensor biases.

### 3.3. Rowing Dead Reckoning (RDR)

**Figure 10:**
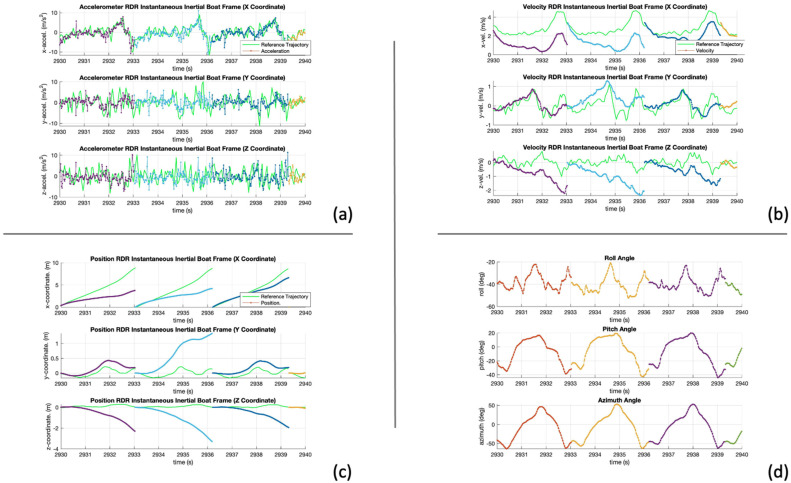
INS RDR results, (**a**) acceleration, (**b**) velocity, (**c**) position, (**d**) attitude in instantaneous inertial boat frame.

RDR is expected to reduce drift errors as each stroke is analyzed independently. Figure 10a shows the acceleration and reference trajectory in the instantaneous inertial XYZ frame. This figure shows that all axes align with the reference trajectory, demonstrating that RDR can reduce the drift from the sensor biases. This is confirmed on Figure 10d; the attitude angles are reset at every stroke, reducing the drift.

Figure 10b shows the velocity, some drift remains affecting all three axes, especially the X- and Z-coordinates. RDR constraints the solution and limits the effect of the biases and errors. However, these biases and errors are not estimated or corrected.

Lastly, the position estimates on Figure 10c show that RDR improves the accuracy of standalone INS without any additional hardware.

### 3.4. INS/GNSS Integration Results

**Figure 11:**
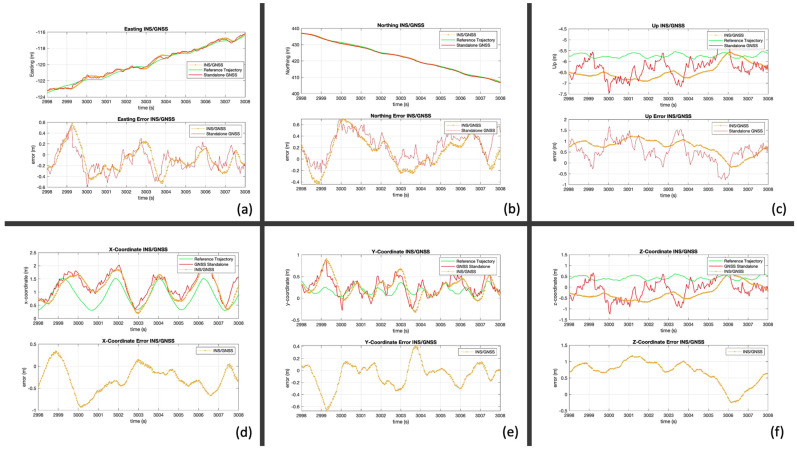
INS/GNSS results. Top ENU frame: (**a**) easting coordinate, (**b**) northing coordinate, (**c**) up coordinate. Bottom boat frame: (**d**) X-coordinate, (**e**) Y-coordinate, (**f**) Z-coordinate.

This section shows the positioning results of the integrated solution from INS and standalone GNSS. On Figure 11, the positions from the standalone GNSS are shown in red, the reference trajectory is in green, and the INS/GNSS solution is in yellow.

First, Figure 11a–c show the position of the handle in ENU. The accuracy in the horizontal plane is below the meter level, which is excellent for navigation. However, the vertical plane is at the meter level. The ENU frame does not provide information about the technique of the rower. Therefore, a transformation into the XYZ frame is needed. Figure 11d–f show the resulting positions. The X- and Y-coordinates provide an estimation of the motion of the handle. However, the accuracy is not sufficient to assess technique. Furthermore, due to the meter-level accuracy of the Z-coordinate, the motion of the handle cannot be observed. It should be noted that the transformation from the ENU to the XYZ frame depends on the two reference GNSS receivers attached to the boat, making this method impractical for a casual user wearing only a GNSS/INS smartwatch, for example.

### 3.5. INS/UWB Integration Results

**Figure 12:**
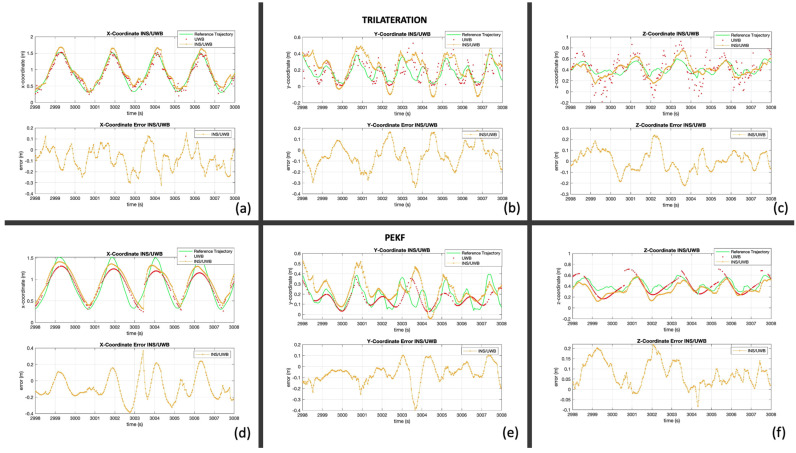
INS/UWB results for boat frame. Top INS/trilateration: (**a**) X-coordinate, (**b**) Y-coordinate, (**c**) Z-coordinate. Bottom INS/PEKF: (**d**) X-coordinate, (**e**) Y-coordinate, (**f**) Z-coordinate.

Trilateration and PEKF demonstrated to be the best models to represent the motion of the handle. Therefore, these are the two models used in the integration. This section shows the results in the XYZ frame. Figure 12 shows the INS/trilateration integration at the top (a–c) and the INS/PEKF integration at the bottom (d–f).

Both aiding sources greatly improved the INS solution and allowed for an accurate representation of the motion of the handle in all axes. These figures demonstrate that UWB can enhance the solution from INS to estimate biases, correct mechanization errors, and allow for technique analysis. However, this method, similar to INS/GNSS, requires the boat reference, provided by two additional GNSS receivers, to transform between frames.

Table 1, Table 2, Table 3 and Table 4 summarize the errors calculated from each method and navigation frame. The error is presented as the mean value and its standard deviation, which represents the accuracy and precision of the solution.

## 4. Conclusions

The WPP demonstrated to be a feasible option for rowing technique analyses because it was able to provide position, velocity, and attitude when INS was integrated with UWB or GNSS. Thus, providing a full understanding of the oar/wrist movement.

UWB trilateration, PEKF, and EKF with constant velocity had total accuracies in the boat frame of ±0.267 m, ±0.187 m, and ±0.270 m, respectively, demonstrating that PEKF was the most accurate UWB standalone positioning method. The integrated methods, INS/GNSS, INS/trilateration UWB, and INS/PEKF UWB obtained accuracies of ±1.386 m, ±0.219 m, and ±0.189 m, respectively, in the boat frame, highlighting that an integrated method provides similar accuracy than standalone UWB with the additional information of velocity and attitude from INS.

In the instantaneous inertial boat frame, integrating INS with RDR had a total accuracy of ±1.656 m. This method was demonstrated to have reduced the effects of sensor biases and reduced accumulated errors. However, it was the least accurate of the integrated methods.

Lastly, in the ENU frame, the integrated methods INS/GNSS, INS/trilateration UWB, and INS/PEKF UWB resulted in accuracies of ±1.375 m, ±0.216 m, and ±0.185 m, respectively, showing that the accuracy of the positioning methods was maintained between navigation frames and that it was possible to obtain the position of the moving boat.

## Figures and Tables

**Table 1:** Sensor specifications of positioning system.

Sensor	Manufacturer	Model	Description	Data Rate	Accuracy
UWB Receiver	Decawave	DW1000	Single-chip wireless transceiver	50 Hz	±10 cm
MEMS IMU	InvenSense	MPU6050	3-axis gyroscope + 3-axis accelerometer	50 Hz	Accel.: 4 g Gyro.: 500 deg/s
GNSS Receiver	U-Blox	ZED-F9P	Multi-band high precision GNSS module	25 Hz	RTK mode: ±0.01 m + 1 ppm CEP
GNSS Antenna	U-Blox	ANN-MB1	Multiband L1/L5	25 Hz	N/A

**Table 2:** Error summary results in boat frame.

3D Outdoor Test Boat Frame Overall (120 s)			
Method	X-axis	Y-axis	Z-axis
	Mean Error-Std. (m)	Mean Error-Std. (m)	Mean Error-Std. (m)
Trilateration UWB	0.023 ± 0.105	−0.015 ± 0.104	0.048 ± 0.222
PEKF UWB	0.022 ± 0.121	−0.020 ± 0.062	0.045 ± 0.129
EKF Const. Vel. UWB	−0.028 ± 0.152	0.002 ± 0.118	−0.025 ± 0.189
INS/GNSS	−0.250 ± 0.458	−0.060 ± 0.503	1.269 ± 1.207
INS/Trilateration UWB	−0.061 ± 0.101	−0.094 ± 0.127	0.103 ± 0.148
INS/PEKF UWB	−0.070 ± 0.117	−0.096 ± 0.106	0.136 ± 0.104

**Table 3:** Error summary results in instantaneous boat frame.

3D Outdoor Test Instantaneous Inertial Boat Frame Overall (120 s)			
Method	X-axis	Y-axis	Z-axis
	Mean Error-Std. (m)	Mean Error-Std. (m)	Mean Error-Std. (m)
INS/RDR	0.172 ± 1.236	−0.583 ± 0.695	0.892 ± 0.856

**Table 4:** Error summary results in ENU frame.

3D Outdoor Test ENU Frame Overall (120 s)			
Method	Easting	Northing	Up
	Mean Error-Std. (m)	Mean Error-Std. (m)	Mean Error-Std. (m)
INS/GNSS	−0.077 ± 0.496	0.131 ± 0.433	1.325 ± 1.207
INS/Trilateration UWB	0.088 ± 0.129	0.077 ± 0.091	0.103 ± 0.148
INS/PEKF UWB	0.089 ± 0.105	0.086 ± 0.111	0.136 ± 0.104

## Data Availability

Raw data from this study can be obtained by contacting the corresponding author.
